# *UNC13D* c.2588G>A Nucleotide Variant Impairs NK-Cell Cytotoxicity in Adult-Onset EBV-Associated Hemophagocytic Lymphohistiocytosis: A Pedigree Study

**DOI:** 10.3390/ijms26178683

**Published:** 2025-09-05

**Authors:** Jia Gu, Ning An, Xinran Wang, Min Xiao, Hui Luo

**Affiliations:** 1 Department of Hematology, Tongji Hospital, Tongji Medical College, Huazhong University of Science and Technology, Wuhan 430030, China; jiagu@tjh.tjmu.edu.cn (J.G.); anning990316@163.com (N.A.); aydwxr428@163.com (X.W.); xiaomin@tjh.tjmu.edu.cn (M.X.); 2 Immunotherapy Research Center for Hematologic Diseases of Hubei Province, Wuhan 430030, China

**Keywords:** *UNC13D*, HLH, EBV, NK-cell cytotoxicity

## Abstract

*UNC13D*, which encodes the Munc13–4 protein, is a critical gene implicated in type 3 familial hemophagocytic lymphohistiocytosis (HLH). While biallelic nucleotide variants in HLH-related genes, including *UNC13D*, are traditionally linked to recessive inheritance patterns in HLH, emerging evidence suggests that heterozygous variants may also contribute to the onset of adult-onset HLH. However, the pathogenicity of heterozygous *UNC13D* variants is still not fully understood. Here, we present a 29-year-old male patient with Epstein–Barr virus (EBV)-triggered adult-onset HLH, who was found to carry compound heterozygous variants in the *UNC13D* gene (c.2588G>A and c.1978_1979insATTACCG) with complete T/NK cytotoxicity dysfunction. We conducted NK-cell function assay in this pedigree to link the genotype to phenotype and demonstrated that the monoallelic *UNC13D* c.2588G>A variant could partially impair NK cell cytotoxicity, in contrast to the completely recessive inheritance observed with *UNC13D* c.1978_1979insATTACCG and other familial HLH-related variants. In addition, to explore the implication of *UNC13D* c.2588G>A variant in various diseases, we reviewed 16 published studies, including data on 35 patients carrying this variant. Data showed the heterozygous variant of *UNC13D* c.2588G>A might act as a genetic risk factor predisposing carriers to conditions like HLH, lymphoma, etc. This study underscores the pathogenic role of the *UNC13D* c.2588G>A variant and expands our understanding of the genetic basis of adult-onset HLH.

## 1. Introduction

HLH represents a severe, life-threatening condition characterized by excessive inflammatory responses, often leading to cytokine storms and multiple organ failure. Without prompt and appropriate management, it can be fatal. The HLH syndrome is often classified into a primary (congenital) form and a secondary (acquired) form. With primary HLH syndrome, the most common form is familial HLH, which is an autosomal recessive syndrome and typically affects children, mostly infants, while the secondary HLH develops following infections, malignancies, and autoimmune diseases, and is much more common in adults. Familial HLH is caused by biallelic variants in four specific genes, *PRF1, UNC13D, STX11*, and *STXBP2* [1,2,3,4], which encode the proteins perforin, Munc13-4, syntaxin-11, and Munc18-2, respectively. These proteins are critical for the cytotoxic activity of NK cells and cytotoxic T lymphocytes (CTLs) and their defect could lead to the dysfunction of NK cells and CTLs [5]. Prior studies have demonstrated that HLH was mechanistically caused by defective lymphocyte cytotoxicity [6].

*UNC13D*, situated on chromosome 17q25 and encoding the Munc13–4 protein, is regarded as a classical gene related to type 3 of familial HLH [7]. *UNC13D* deficiency accounts for 30–35% of familial HLH cases [8]. A pivotal role of Munc13–4 protein is to initiate the degranulation process in NK cells and CTLs. When NK cells or CTLs recognize and bind to infected cells, the Munc13-4 protein mediates the fusion of cytotoxic granules (containing perforin and granzymes) with the cell membrane, facilitating the release of granular contents to extracellular space to eliminate target cells [6]. Variants in this gene may lead to dysfunction of NK cells and CTLs, impairing their ability to effectively clear infected cells and subsequently triggering excessive inflammatory responses.

Recent studies have shown that heterozygous variants of familial HLH genes were associated with the development of adult-onset HLH, suggesting that the inheritance mechanism for these conditions is not incompletely recessive [2,9,10]. A previous report found that monoallelic *STXBP2* variant affecting codon 65 compromised lymphocyte cytotoxicity dominantly in a negative manner to impair membrane fusion and arrest SNARE-complex assembly [11]. In fact, the implication of most heterozygous variants of HLH genes was uncertain and was hard to verify.

A previous cell-based functional assay, which assessed the function of *UNC13D* gene by the knocking out of the mouse orthologue of the human *UNC13D* gene in CD8+ T cells in mice and simultaneous replacement of this gene with human gene *UNC13D* carrying c.2588G>A variant, demonstrated that *UNC13D* c.2588G>A variant exerted no cytotoxicity on day 4 in *UNC13D* knockout cells, but the activity was partially restored by day 7 [12], suggesting complex pathogenicity of this nucleotide variant. Although the heterozygous *UNC13D* c.2588G>A nucleotide variant is frequently associated with HLH and refractory viral infections [13,14,15], how this monoallelic variant contributes to disease pathogenesis remains uncertain. In this study, we reported an adult HLH patient with EBV infection carrying *UNC13D* c.2588G>A and reviewed this variant in previous studies, aiming at looking into the role of this monoallelic variant in diseases.

## 2. Detailed Case Presentation

### 2.1. Patient, Clinical Presentation and Diagnosis

The 29-year-old Han-nationality Chinese male was admitted to our hospital due to recurrent fever lasting 2 months, thrombocytopenia, and anemia. The patient had no significant medical history. At disease onset, the patient presented to a local hospital with hepatosplenomegaly and pancytopenia (white blood cell [WBC] count, 2.52 × 10^9^/L; hemoglobin, 74 g/L; platelet count, 7 × 10^9^/L); hypofibrinogenemia (1.16 g/L); EBV-DNA in PBMCs (5.62 × 10^4^ copies/mL); ferritin (30,477.8 μg/L); soluble interleukin-2 receptor [sIL-2R] level > 7500 U/mL; and mild elevation of liver enzymes (alanine aminotransferase 64 U/L, aspartate aminotransferase 59 U/L). Bone marrow examination confirmed hemophagocytosis. These findings met 7/8 HLH-2004 diagnostic criteria [16] (fever, hepatosplenomegaly, cytopenias, hyperferritinemia, hypofibrinogenemia, hemophagocytosis, elevated sIL-2R) and the HScore [17] for the diagnosis of reactive hemophagocytic syndrome was 239, corresponding to a probability of over 98% for hemophagocytic syndrome.

**Figure 1:**
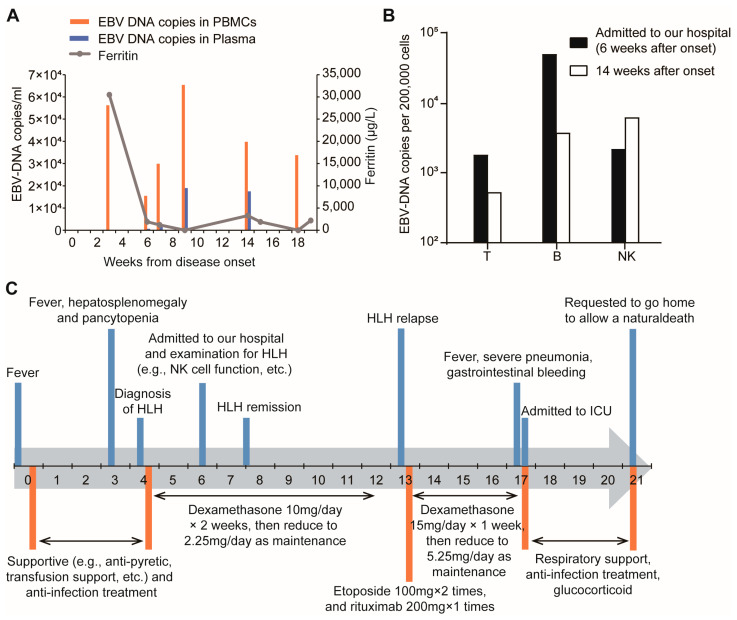
EBV DNA detection and clinical timeline of the patient. (**A**) Serum ferritin level, EBV DNA loads in peripheral blood mononuclear cells (PBMCs) and plasma during the disease course. (**B**) EBV DNA loads in T cells, B cells, and NK cells at 6 and 14 weeks after disease onset. (**C**) Timeline of the patient’s diagnosis and treatment.

At 6 weeks after disease onset, the patient was admitted to our hospital. Further analysis of EBV DNA levels showed significant alterations in both plasma and PBMCs (Figure 1A). Subsequent investigation on EBV-infected cells identified B cells as the primary target (5.093 × 10^4^ copies per 2 × 10^5^ B cells, 2.193 × 10^3^ copies per 2 × 10^5^ NK cells, and 1.796 × 10^3^ copies per 2 × 10^5^ T cells) (Figure 1B). EBV serological test data were unavailable for this patient, precluding a definitive classification as primary infection or reactivation. Nonetheless, as the disease progressed, NK cells and T cells became increasingly involved (Figure 1B). In addition, we ruled out other infections, tumor-related diseases, autoimmune and rheumatic disorders through PET-CT, bone marrow biopsy, and a comprehensive rheumatological panel. Ultimately, the patient was diagnosed as having EBV-triggered adult-onset primary HLH.

### 2.2. Genetic Testing

**Figure 2:**
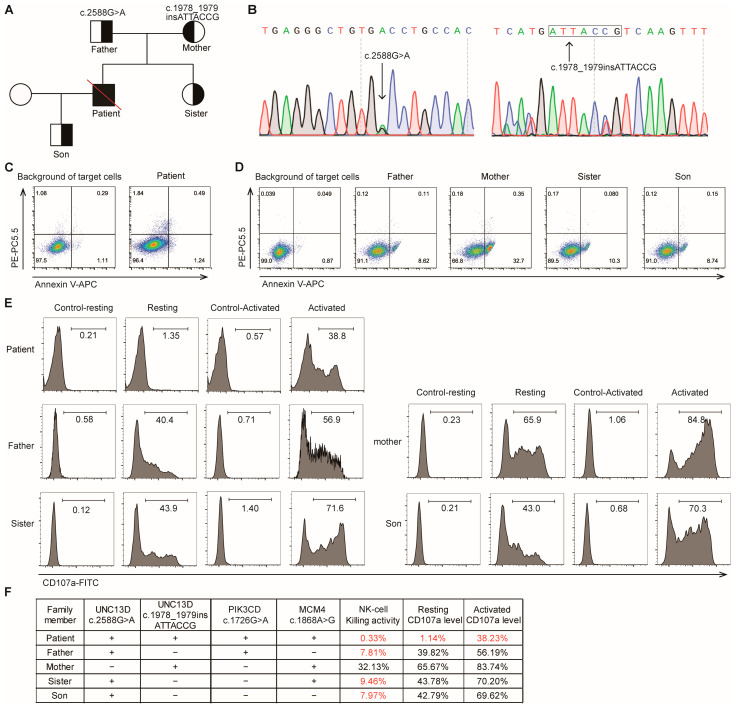
Genetic features and NK-cell cytotoxic function in the patient and his family members. (**A**) Pedigree analysis of the patient with *UNC13D* nucleotide variants. (**B**) *UNC13D* nucleotide variants was verified by Sanger sequencing. (**C**,**D**) NK-cell killing activity was assessed by flow cytometry. The NK-cell killing activity = Early apoptotic and necrotic cells (%) in experimental group (patient)—Early apoptotic and necrotic cells (%) in control group (background of untreated target cells). The cell population in the upper right quadrant (AnnexinV-APC+ PE-PC5.5+) represents necrotic cells, and the cell population in the lower right quadrant (AnnexinV-APC+ PE-PC5.5−) represents early apoptotic cells. (**E**) Expression level of resting and activated CD107a (a marker of NK cell degranulation) was flow cytometrically determined. Resting CD107a level in NK cells = CD107a expression (%) in resting group—CD107a expression (%) in control-resting group; Activated CD107a level in NK cells = CD107a expression (%) in activated group—CD107a expression (%) in control-activated group. (**F**) Summarization of genetic features, NK-cell killing activity, and CD107a expression in the patient and his family members. Text in red denotes impaired NK cell function; text in black denotes normal function.

Whole-exome sequencing (WES) screening for HLH-related nucleotide variants yielded four genetic variants in *UNC13D*, *PIK3CD*, and *MCM4* genes, which were subsequently confirmed in the family (Figure 2F). Among these, a missense variant (c.2588G>A) and a frameshift insertion (c.1978_1979insATTACCG) were identified in the *UNC13D* gene, along with several variants of uncertain significance in the context of HLH (Figure 2B). Two-generation pedigree analysis using Sanger sequencing demonstrated that these variants were inherited from the patient’s parents, with the patient’s sister and son also inheriting the *UNC13D* c.2588G>A variant, respectively, from their father (Figure 2A).

### 2.3. NK Cell Cytotoxicity Assays

To assess the impact of the *UNC13D* variants on T/NK cell cytotoxicity, NK cell killing assays were performed on the patient and his family members by referring to previous studies. NK cell killing activity in the patient carrying the compound heterozygous *UNC13D* c.2588G>A and c.1978_1979insATTACCG variants was markedly lowered to 0.33% (normal ≥15.11%) [18] (Figure 2C), and the expression of resting CD107a (1.14% [normal ≥ 5%]) and activated CD107a (38.23% [normal ≥ 40%]), a marker for NK cell degranulation [5,10], dropped significantly (Figure 2E). Specifically, the patient’s father, who also carried the c.2588G>A variant, and his sister and son, who inherited the nucleotide variant from their father, exhibited reduced NK cell cytotoxicity and had lower resting and activated CD107a expression in NK cells compared to the patient’s mother (Figure 2F). The patient’s mother, carrying the c.1978_1979insATTACCG variant, showed normal NK cell function (Figure 2D,E). The results revealed that the heterozygous variant c.2588G>A could partially impair NK cell function, while the monoallelic c.1978_1979insATTACCG variant had no impact on NK cell cytotoxicity. The sequencing results and the profile of NK cell function of the patient’s family are shown in Figure 2F.

### 2.4. Treatment and Disease Progression

Upon diagnosis, the patient received dexamethasone 10 mg/day for approximately 2 weeks at a local hospital and dexamethasone was gradually reduced to 2.25 mg/day as maintenance dose to prevent recurrence of HLH. Initially, the disease was well-controlled, with the ferritin level dropping from 30,477.8 to 1199.1 μg/L (Figure 1A) and his body temperature being normal. However, his condition was out of control one month later. The patient developed recurrent high-grade fever, and the level of his inflammatory factors was elevated, with plasma EBV-DNA at 3.98 × 10^4^ copies/L and EBV-DNA in PBMCs at 1.75 × 10^4^ copies/L (Figure 1A). At 14 weeks after his disease onset, the patient developed a recurrence of fever 1 week after self-discontinuation of glucocorticoid, suggesting a relapse of HLH. The results of EBV-DNA sorting PCR suggested the predominant EBV infected cells were NK cells (3.815 × 10^4^ copies per 2 × 10^5^ NK cells, 5.29 × 10^3^ copies per 2 × 10^5^ T cells, and 3.815 × 10^3^ copies per 2 × 10^5^ B cells) (Figure 1B). We switched his therapy to the HLH-94 protocol consisting of etoposide, dexamethasone, and rituximab. Then, his ferritin levels declined from 3298.7 μg/L to 1878.2 μg/L and the platelet count increased from 31 × 10^9^/L to 91 × 10^9^/L. Three weeks later, the patient experienced a recurrence of fever, accompanied by severe pulmonary infection, decreased blood oxygen saturation, liver dysfunction, and thrombocytopenia. Despite the symptomatic treatments, including anti-infection therapy and corticosteroids, the patient’s condition did not improve. Subsequently, the patient was transferred to the intensive care unit (ICU) due to shock and further decline in blood oxygen saturation, as depicted in the timeline in Figure 1C. The patient’s condition continued to deteriorate, necessitating the use of high-dose vasopressors to maintain blood pressure and high mechanical ventilation to support oxygen saturation. On day 104 following the diagnosis, the patient’s family requested his transfer home to die in palliative care.

### 2.5. Literature Review

To explore the significance of *UNC13D* c.2588G>A variant in various diseases, we reviewed the existing literature regarding this variant. We synthesized findings from 16 published studies, including data on 35 patients carrying this variant (Table 1). Among the 35 patients identified, the majority (*n* = 22) were diagnosed with HLH. The most common genotype in these HLH patients was compound heterozygous (45.4%, 10/22), followed by heterozygous (27.3%, 6/22) and homozygous (27.3%, 6/22). The second most common diagnosis was lymphoma (*n* = 9), with most cases being heterozygous (*n* = 7). Additionally, four patients were diagnosed as having autoimmune lymphoproliferative syndrome (ALPS), macrophage activation syndrome (MAS), or severe COVID-19, with nucleotide variants being homozygous or compound heterozygous.

## 3. Discussion

In this case report, we presented an adult patient with compound heterozygous *UNC13D* variants: a missense variant (c.2588G>A) and a frameshift insertion (c.1978_1979insATTACCG). We also conducted NK cell function assay in this pedigree to link the genotype to phenotype. We demonstrated that the monoallelic *UNC13D* c.2588G>A variant could partially impair NK cell cytotoxicity, in contrast to the completely recessive inheritance observed with *UNC13D* c.1978_1979insATTACCG and other familial HLH-related variants. Moreover, we reviewed the existing literature on *UNC13D* c.2588G>A and found that this variant was frequently associated with a number of diseases, including HLH, lymphomas, MAS, and ALPS. These findings suggest that the variant might be linked to the development of lymphoproliferative and immune-related diseases. Although larger cohort studies and functional assays are essential to clarify the contribution of this variant to disease pathogenesis, multiple previous studies have verified that haploinsufficiency of *UNC13D* increased the risk for lymphoma or extreme high hyperinflammatory status after infection [33,34]. Among these diseases, most patients had a later-onset of symptoms, and many carried the heterozygous variants. On the basis of these findings, we are led to theorize that this variant might be of conditionally pathogenic nature, and was triggered by factors such as viral infections, combination with other nucleotide variants, or age-related decline in immune function.

In the general population, *UNC13D* c.2588G>A variant is not rare, with a frequency of up to 0.36% in East Asian populations of Genome Aggregation Database (GenomeAD) exome cohort [35]. Integrating its recurrence in HLH cases (including our patient) and a previous function study [12], we conclude that the variant functions as a hypomorphic allele requiring co-factors for phenotypic expression in heterozygotes. The 0.36% prevalence does not amount to clinically relevant NK dysfunction in carriers, as cytotoxicity impairment manifests primarily under concurrent triggers.

This patient carried not only *UNC13D* variants, but also *PIK3CD* and *MCM4* variants. As previously reported [36], *MCM4* deficiency was shown to impair NK cell function and primarily reduce the number of mature NK cells. However, our patient exhibited normal NK cell counts. Additionally, the patient’s mother (heterozygous carrier of *MCM4* c. 1868A>G) had no past history of viral infections and her NK cell function was fully preserved. Therefore, the *MCM4* variant lacks clinical evidence for its pathogenicity. Furthermore, we predicted the functional impact of this *MCM4* and *PIK3CD* variants using bioinformatic tools (https://varsome.com/, accessed on 11 August 2025). The in silico tool predicted a benign outcome with both variants. No clinical diagnostic laboratories have submitted clinical-significance assessments for these variants to ClinVar. Based on these evidence, we identified *UNC13D* variants as the primary contributor to the patient’s disease, outweigh *MCM4*/*PIK3CD* variants.

It is worth noting that, in clinical practice, monoallelic *UNC13D* c.2588G>A variant is potentially pathogenetic in some conditions and is intimately associated with the occurrence of many diseases. Although our study is conducive to the understanding of the role of *UNC13D* c.2588G>A, NK cell cytotoxicity assay is affected by many factors, and the influence of *UNC13D* c.2588G>A variant on the NK cell function needs to be verified in more healthy volunteers carrying this variant.

## 4. Materials and Methods

### 4.1. NK Cell Killing Activity Analysis

Peripheral blood mononuclear cells (PBMCs) were isolated from peripheral blood samples using the Ficoll-Hypaque density gradient centrifugation and resuspended at 5 × 10^6^/mL. They were then co-cultured with K562 cells expressing EGFP flag at a 10:1 ratio for 4 h. The K562 cells cultured alone were used as the control group. Apoptosis of K562 target cells was evaluated by using Annexin V-APC and propidium iodide (PI) staining. The NK cell killing activity = Early apoptotic and necrotic cells (%) in experimental group—Early apoptotic and necrotic cells (%) in control group. The established normal range was derived from previous studies and validated in our preliminary studies using healthy donors [18].

### 4.2. NK Cell CD107a Degranulation Assay

Expression level of resting and activated CD107a expression level of resting and activated CD107a (a marker of NK cell degranulation) was flow cytometrically determined. PBMCs were divided into four groups (resting-control group: PBMCs; resting group: PBMCs + K562; activated-control group: PBMCs + IL-2 100IU/mL; activated group: PBMCs + IL-2 100IU/mL + K562). PBMCs were resuspended at 2 × 10^6^/mL and co-cultured with K562 cells at a 1:1 ratio for 2 h. To each well, 0.5 μL of monensin solution and 2 μL of FITC-conjugated anti-CD107a antibody were added for protein transport inhibition and degranulation detection. Following incubation, the cells were collected, stained with anti-CD45-PE, anti-CD3-PerCP and anti-CD56-APC antibodies, and flow cytometrically analyzed to assess CD107a expression on CD3−CD56+ NK cells. Resting CD107a level in NK cells = CD107a expression (%) in resting group—CD107a expression (%) in resting-control group; Activated CD107a level in NK cells = CD107a expression (%) in activated group—CD107a expression (%) in activated-control group. The established normal range is derived from previous studies and validated in our preliminary studies using healthy donors [5,10].

### 4.3. EBV-DNA Sorting PCR

PBMCs were isolated from peripheral blood via density gradient centrifugation (TBD, Tianjin, China), counted on a hemocytometer, and magnetically sorted using immunomagnetic beads (Miltenyi, Bergisch Gladbach, Germany) to obtain CD3+ T cells, CD19+ B cells, and CD3−CD56+ NK cells. Purities of sorted cells were confirmed by flow cytometry (97–99% for B/T cells and 91–95% for NK cells). Then, purified cells were counted, and 2 × 10^5^ cells were analyzed by subsequent quantitative PCR as previously described [37].

### 4.4. Whole-Exome Sequencing Analysis

Genomic DNA was extracted using the QIAamp DNA Blood Mini Kit (Qiagen, Hilden, Germany). An amount of 500 ng of extracted genomic DNA was randomly sheared into 150–250 bp fragments using ultrasonication. The whole-genome sequence library was constructed using TargetSeq^®^ hybrid capture sequencing technology (iGeneTech, Beijing, China) and sequenced with 150 bp paired-end reads on the NextSeq 550 platform (Illumina, San Diego, CA, USA). For bioinformatic analysis, low-quality data were filtered using FastQC (v0.11.7), sequence alignment was performed with BWA (reference genome: GRCh37/hg19), and variant annotation was conducted using GATK (The Genome Analysis Toolkit, v3.8-1-0, URL: https://software.broadinstitute.org/gatk/gatk3, accessed on 11 August 2025).

## 5. Conclusions

This study underscored the pathogenic role of the *UNC13D* c.2588G>A variant and expanded our understanding of the genetic basis of adult-onset HLH. Through this pedigree study, we demonstrated that the monoallelic *UNC13D* c.2588G>A variant could partially impair NK cell cytotoxicity, in contrast to the completely recessive inheritance observed with *UNC13D* c.1978_1979insATTACCG and other familial HLH-related variants. In addition, we reviewed 16 published studies, including data on 35 patients carrying this variant. Data showed that the heterozygous variant of *UNC13D* c.2588G>A might act as a genetic risk factor predisposing carriers to diseases like HLH, lymphoma, etc.

## Figures and Tables

**Table 1:** Summary of diseases caused by the *UNC13D* c.2588G>A variant in previous reports.

References	Patients	Sex	Age of Onset	Diseases	Zygosity	EBV Infection
[14]	P1	Female	54 years	NK/T-NHL	Heterozygous	NA
[14]	P2	Male	46 years	NHL	Heterozygous	NA
[14]	P3	Female	12 years	NHL	Heterozygous	NA
[14]	P4	Male	40 years	B-NHL	Heterozygous	NA
[14]	P5	Female	30 years	NK/T-NHL	Heterozygous	NA
[14]	P6	Male	28 years	NHL	Heterozygous	NA
[14]	P7	Male	9 years	HL	Homozygous	+
[14]	P8	Female	54 years	NK/T-NHL	Heterozygous	NA
[19]	P9	Male	13 years	HLH	Compound heterozygous	NA
[19]	P10	Male	15 years	HLH	Homozygous	NA
[20]	P11	Female	52 years	HLH	Homozygous	+
[21]	P12	Female	64 years	Severe COVID-19	Compound heterozygous	NA
[22]	P13	Male	10 years	ALPS	Homozygous	+
[23]	P14	Male	13 years	HL	Homozygote	NA
[23]	P15	Female	27 years	HLH	Homozygote	NA
[23]	P16	Male	35 years	HLH	Homozygote	NA
[23]	P17	Male	52 years	HLH	Homozygote	NA
[23]	P18	Female	29 years	HLH	Heterozygous	NA
[23]	P19	Male	5 years	HLH	Heterozygous	NA
[23]	P20	Male	31 years	HLH	Heterozygous	NA
[24]	P21	NA	NA	MAS	homozygote	NA
[25]	P22	Male	38 years	HLH	Compound heterozygous	−
[26]	P23	NA	NA	HLH	Compound heterozygous	NA
[26]	P24	NA	NA	HLH	Heterozygous	NA
[26]	P25	NA	NA	HLH	Compound heterozygous	NA
[27]	P26	Female	9 years	HLH	Compound heterozygous	NA
[28]	P27	Male	1 month	HLH	Compound heterozygous	−
[29]	P28	Female	7 years	CNS-HLH	Compound heterozygous	−
[29]	P29	Female	NA	CNS-HLH	Compound heterozygous	−
[30]	P30	Male	15 years	MAS	Homozygous	NA
[20]	P31	Male	52 years	HLH	Homozygous	+
[31]	P32	Male	2 years	HLH	Heterozygous	NA
[31]	P33	Male	3 years	HLH	Heterozygous	NA
[32]	P34	Female	18 years	HLH	Compound heterozygous	+
[32]	P35	Male	21 years	HLH	Compound heterozygous	+

ALPS, autoimmune lymphoproliferative syndrome; COVID-19, coronavirus disease 2019; HL, Hodgkin lymphoma; HLH, hemophagocytic lymphohistiocysis; MAS, macrophage activation syndrome; NA, not applicable; NHL, non-Hodgkin lymphoma; NK/T, natural killer/T-cell; +, positive; −, negative; NA, unavailable.

## Data Availability

Original data are available from the corresponding author upon reasonable request.

## Abbreviations

NK	nature killer
CTLs	cytotoxic T lymphocytes
EBV	Epstein–Barr virus
HLH	hemophagocytic lymphohistiocytosis
WBC	white blood cell
DNA	Multidisciplinary Digital Publishing Institute
PBMCs	peripheral blood mononuclear cells
IL-2	interleukin-2
sIL-2R	soluble interleukin-2 receptor
PCR	polymerase chain reaction
PET-CT	positron emission tomography/computed tomography
WES	whole-exome sequencing
ICU	intensive care unit
ALPS	autoimmune lymphoproliferative syndrome
MAS	macrophage activation syndrome
CD	cluster of differentiation
COVID	coronavirus disease
EGFP	enhanced green fluorescent protein
APC	allophycocyanin
PI	propidium iodide
PerCP	Peridinin-Chlorophyll-Protein
